# 

**Published:** 1842-09-24

**Authors:** 


**CONTENTS OF No. 39.**

Dr. Pancoast's Case of Excision of the Elbow-Joint, - - - - - 609

Dr. Bolton's Cases of Foreign Bodies removed from the Ear and Esophagus, - - - - - - - - - - - - - 613

BIBLIOGRAPHICAL NOTICES: Animal Chemistry, or Organic Chemistry in its applications to Physiology and Pathology. By Justus Liebig, M. D., &c. Professor of Chemistry in the University of Giessen. Edited from the author's manuscript by William Gregory, M. D., &c. - - - - - - - 614

Case of Ununited Fracture of the Forearm of four years standing, successfully treated. By Charles S. Tripler, M. D., Surgeon U. S. Army, - - - - - - - - - 618

EDITORIAL:—Miscellaneous Notices.

University of Virginia, - - - - - - - - 619

Medical College of Missouri, - - - - - - - 619

New Haven Medical Association, - - - - - - 619

ANALECTA: Dr. Bartlett's Case of Typhoid Fever in a patient sixty-three years old, - - - - - - - - - - - 620

Dr. Markwick on Rhubarb as an application to Ulcerated Surfaces, - - - - - - - - - - - - - 622

Dr. Green's Case of Amputation of the Leg, with some Observations on a New Mode of Amputating, - - - - 623

